# Assessment of the impact of shaped nozzles installed inside the pipeline on the energy efficiency of compressed gas systems

**DOI:** 10.1038/s41598-023-43620-x

**Published:** 2023-09-29

**Authors:** Przemysław Młynarczyk, Damian Brewczyński, Joanna Krajewska-Śpiewak, Kamil Chmielarczyk, Jarosław Błądek, Paweł Lempa

**Affiliations:** Faculty of Mechanical Engineering, Cracow University of Technology, al. Jana Pawla II 37, 31-864 Krakow, Malopolskie Poland

**Keywords:** Natural gas, Mechanical engineering

## Abstract

Pressure pulsations and vibrations generated in gas discharge pipelines are one of the main causes of failure in a compressed gas system. Installation of shaped nozzles in the compressor discharge manifold is one of the new ideas to minimize this phenomenon. It has been proven that shaped nozzles technology is able to minimize the unfavorable phenomena of pressure pulsation and thus the pipeline vibration. The production of such components using 3D printing techniques is a very good solution, as they have complicated shapes and are individually produced for a specific installation. The world is currently struggling with an excess of waste and a shortage of energy. Therefore, modern technology should be part of the sustainable development strategy, according to which the amount of energy consumed during the processes should be reduced. This article presents the influence of shaped nozzles on the specific compression power mounted in the discharge manifolds of two different compressors: reciprocating and screw. This influence can also be estimated by a conceptual model presented in the article. Based on the values of specific compression power, obtained during carried out research, it can be concluded that 3D printed nozzles may have a minor impact on the energy efficiency of compression depending on their shape complexity.

## Introduction

Pressure pulsations and pipeline vibrations are the main phenomena that influence the risk of failure of gas installations. Various methods have been used to reduce the phenomenon in the past. Currently, a new method is in use where the nozzles are mounted inside the pipeline. This new approach differs significantly from the widely used Helmholtz resonators. In the case of damping nozzles, not acoustic but flow phenomena may be the main factor affecting the damping of pressure pulsations^1^. All applied methods have an influence on the specific compression power (SCP). Thanks to the use of 3D printing, the shape of the nozzles can be optimized so that the energy consumption is not significantly increased with the simultaneous damping of pulsations. At the 7th Annual Global Conference on Energy Efficiency, it was agreed that the current operating strategy should initially focus on increasing the efficiency of existing installations^2^. The paper^3^ describes that compressed air accounts for up to 10% of industrial electricity consumption in the European Union, 9.4% of China’s electricity, and in the United States, compressed air systems account for about 10% of total industrial energy use and in South Africa compressed air consumes about 9%. Therefore, issues related to the topic of energy optimization in compressor systems are a very popular topic widely described in the scientific literature. In paper^4^ the authors describe how important the efficiency improvement of the compressors used in compressed air energy storage is. The authors propose a novel method which could improve compressor efficiency by improving the heat transfer in the compressor piston making the compression process near isothermal. Authors of the publication^5^ prepare a special model which can help predict the specific energy consumption by compressors in manufacturing compressed air systems with dynamic energy consumption profiles. Optimization of the axial flow compressor in terms of efficiency for Brayton cycle plants is presented in^6^. Dos Santos Mascarenhas et al.^7^ present extensive energy, exergy, sustainability and emission analysis of industrial air compressors. They conclude their work that the main areas where energy savings can be done are the reduction of discharge pressure, improvement of power factor and waste heat recovery. Not only the compressor system itself influences the overall efficiency. In refrigeration systems the coolant which is used also has a high impact on the compressing process efficiency. Such analysis is presented in the paper^8^. Nowadays hydrogen is becoming a fuel of the future, therefore, research regarding transporting and storage of this medium is very important. In the paper^9^ the technology of introducing a porous media plate into the hydrogen compressor piston is presented. Such technology influences compressor efficiency and gives heat savings for the revolution speed specified by the authors. As compressors are widely used machines, the authors of paper^10^ investigate the influence of compressor efficiency on the environmental impact in wastewater plant. Investigations where the condition of the gas at the intake pipe influences the compression efficiency in the example of a wet compression system can be also found in the scientific literature^11^. In particular, the article^12^ presents considerations related to the energy efficiency of a system with variable compressor frequency. The main energy savings in compressed-air systems are: eliminating leakages, minimizing pressure drop, heat recovery and optimization of drive systems and the overall process^13^. Thus, the new technology must have no or minimal impact on the increase in compression power. This paper proposes a simple predictive model of the effect of complex shaped nozzles in the manifold on the specific compression power. Such a model will allow us to assess the suitability of the nozzle for use and, at the same time, save the material that would have been used to manufacture the test components. Producing nozzles with the use of 3D printing not only freedom in designing but also reduces production waste. These methods have also been known for years and are used in both retail and mass production. Issues related to proper management of raw materials and the impact of production methods on the environment are increasingly analyzed in the scientific literature^14^. The authors^15^ presented the similarities, advantages and disadvantages of additive manufacturing (AM) and conventional methods (CM) taking into account economic, ecological (environmental) and technological aspects. The analysis shows that in the case of large-scale and mass production, traditional manufacturing methods should still be used. Additive methods are more flexible for the production of personalized components and parts with complex geometries. This situation can be encountered when designing nozzles for a specific gas installation.

## Geometry of tested nozzles

The main purpose of this study is to evaluate the effect of nozzles mounted inside the pipeline, on the specific compression power in the displacement compressor discharge system. As the nozzles of the presented shapes had a beneficial effect on suppressing pressure pulsations in compressor systems^16^ an adequate evaluation of their effect on compression power is necessary. The paper is mainly focused on a comparative analysis needed to determine the direction in which the geometry of such elements should be developed. In order to obtain a reliable comparison, nozzles with more and less complex shapes were considered.

**Figure 1:**
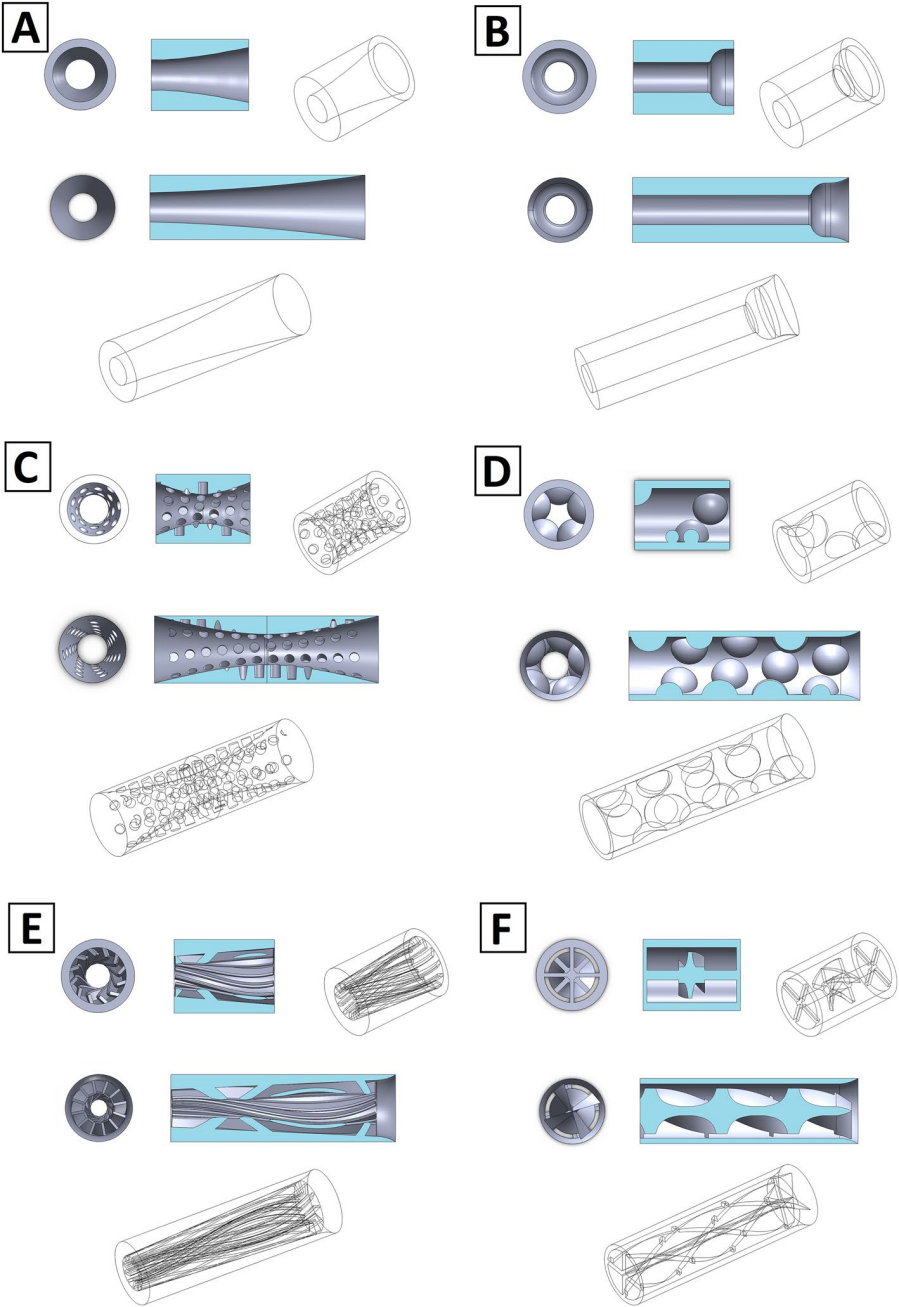
Shapes of the investigated nozzles. (**A**) hyperboloidal shape, (**B**) brass instrument mouthpiece shape, (**C**) double hyperboloidal shape with cavities, (**D**) circumferential hemispheres shape, (**E**) ribbed shape, (**F**) turbine-like shape.

In the presented analysis, the selection of shapes was made on the basis of various geometrical concepts. Then, the impact of the damper assembly on the compression power was assessed, which allowed the design direction in which the technology should be developed. Figure 1 shows 3D models of all analyzed nozzles for reciprocating and screw compressor test stands. The preliminary influence on the pressure pulsations and pipeline vibration of these nozzles, in the reciprocating compressor installation, was presented in the paper^16^ and is still evaluated.

All elements were designed to meet a condition in which the change of the gas flow surface area in the passage is equal to 70% of the flow area of the pipe in which the elements are mounted. The installation with an element that maintains the diameter of the pipeline was a reference point for the results obtained for various nozzle shapes. Later in this article, this type of installation is referred to as an empty pipe. On the basis of previous tests, the large value of the constriction was selected, to emphasize the significant impact of the elements on the pulsation and vibration damping and especially on the specific compression power. The results, regarding the increase in power demand may, in this case, be more unfavorable than when the necessary constriction value is optimized.

The research on the damping of pressure pulsations, allowed to conclude that nozzles with a hyperboloidal shape suppress pressure pulsations well. At the same time they do not cause a large increase in compression power^1,17,18^. For this reason, this shape of the printed nozzle was chosen for comparison with other, more complex shapes. The B-shape nozzle is based on the shape of the mouthpiece for brass instruments. This shape is characterized by the achievement of the maximum assumed flow restriction over a very short section, which can significantly affect the flow resistance, the radius of the “bowl” corresponds to 1/6 of the inlet diameter of the nozzle. C-shape nozzle is a double hyperboloid nozzle with extra holes along the length of the nozzle. These holes are additional places of stream continuity disturbance and have an impact on the pulsations damping by the generation of a large number of small vortices. D-shape nozzle is a tube with circumferential hemispheres along the entire length of the nozzle. It is a shape where the volume of fluid inside the nozzle is significant. There are no sharp edges in the geometry of the material that could cause significant vortices. The hemispheres are designed to have the base tangential to the inner surface of the damper cylinder. E-shape nozzle is a linearly tapering, internally ribbed nozzle shape, based on the design of a threaded gun barrel. The ribs are curved at about 5 degrees and the height of the ribs is 1/6D. Therefore, an additional surface is created that generates resistance in the direction of fluid flow. This design, in option, allows the element to rotate through the flowing gas, but it is not the subject of this paper. The F-shape nozzle is a turbine-like shape, and in the case of a larger nozzle, this shape is multiplied in a linear pattern. The shape of the nozzle was achieved by rotating the cross along the axis of the nozzle, and then the generated shape was sharpened. This shape can also be used in a rotating form in the future.

## Determination of the specific compression power

The basic quantity describing the efficiency of compression is the Specific Compression Power (SCP)^19^. The determination of the specific compression power can be expressed in two ways: by describing the amount of kg or m^3^ (under standard conditions) of gas compressed using 1 kW of energy, or by describing how many kW of energy is consumed to compress 1 kg or m^3^ . In this paper, the first definition is adopted. The nozzles were tested on two test stands. During the tests, the values related to the assessment of the impact of nozzles on the energy efficiency of a given installation were measured. The source of the pulsating flow in the first test stand was a reciprocating compressor (SAF-23), which generates low frequencies of pressure pulsation. A DEMAG.DS-40 CompAir—screw compressor was used on the second test stand, which allows to achieve higher discharge pressure and higher frequencies of pressure pulsation. All tests, on both test stands, were carried out according to the well-established procedure and with full control of the ambient conditions. The average temperature of the intake air during the experiments was 25 ± 2 °C.

### The two-cylinder reciprocating air compressor

**Figure 2:**
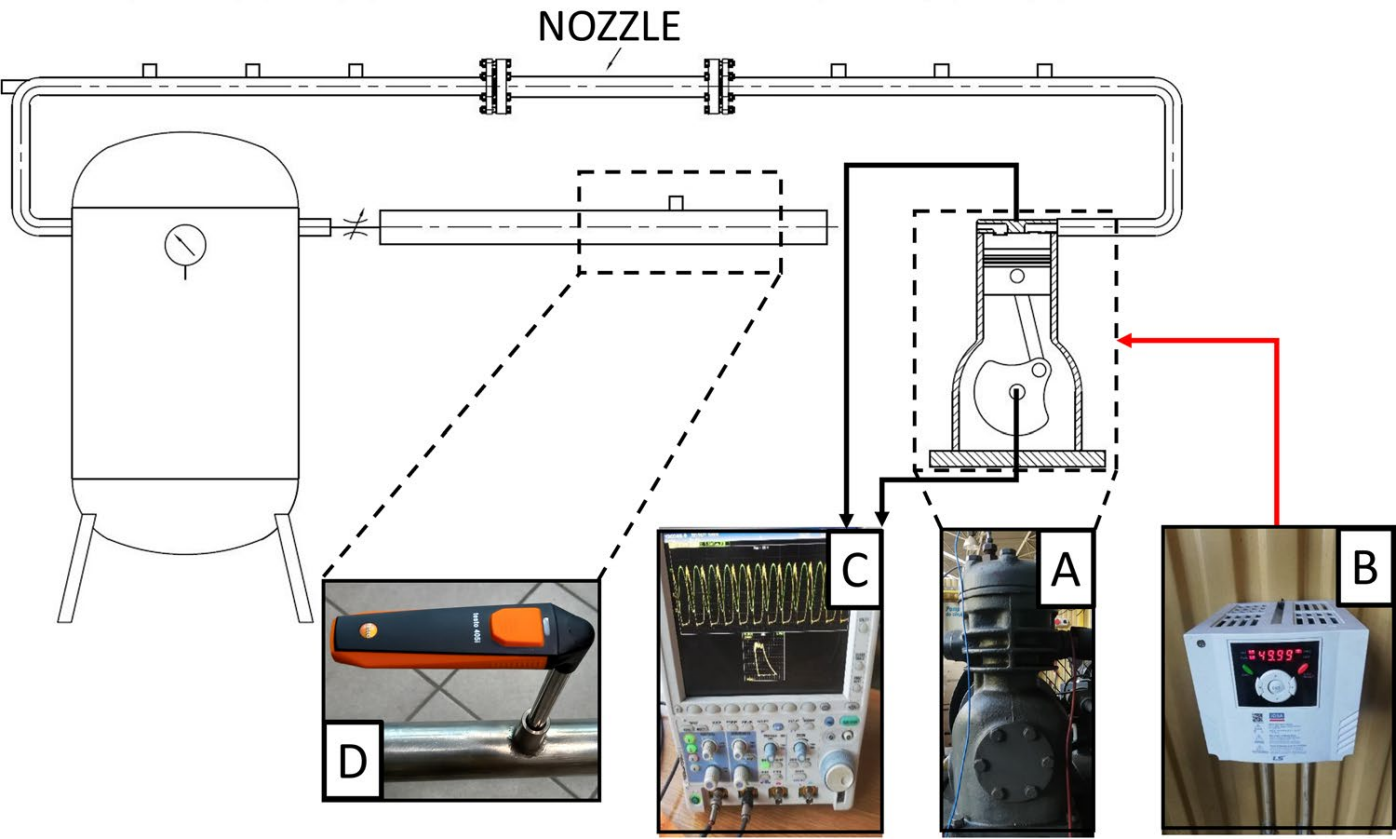
The scheme of the test stand with reciprocating compressor. (**A**) Compressor, (**B**) Inverter, (**C**) Osciloscope, (**D**) Thermoanemometer.

The two-cylinder reciprocating air compressor is driven by a variable-speed electric motor and an inverter was used to control the rotational speed of the drive shaft. For all measurements, the discharge pressure was kept constant at 150 kPa. Measurements were performed for two different revolution speeds: 720 and 1080 revs/min. For each case the mass flow rate was calculated with the use of temperature and flow velocity measured by the thermoanemometer. The compression power was calculated from the indicator chart. The test stand scheme is shown in Fig. 2. Compressor outlet pipe has an inner diameter of 12 mm, installation pipe diameter is 22 mm and the diameter of nozzle mounting pipe is 17 mm. The total length of the installation from the compressor to the tank is about 4 m. Compressor shaft revolution speed was measured with the Hasler analogue tachometer with reading accuracy + /− 2 rpm. The volumetric flow rate was calculated using readings from the Testo 405i thermoanemometer with an accuracy of ± 0.1 m/s + 5% of measured velocity (for gas velocity) and ± 0.5 °C for temperature.

**Figure 3:**
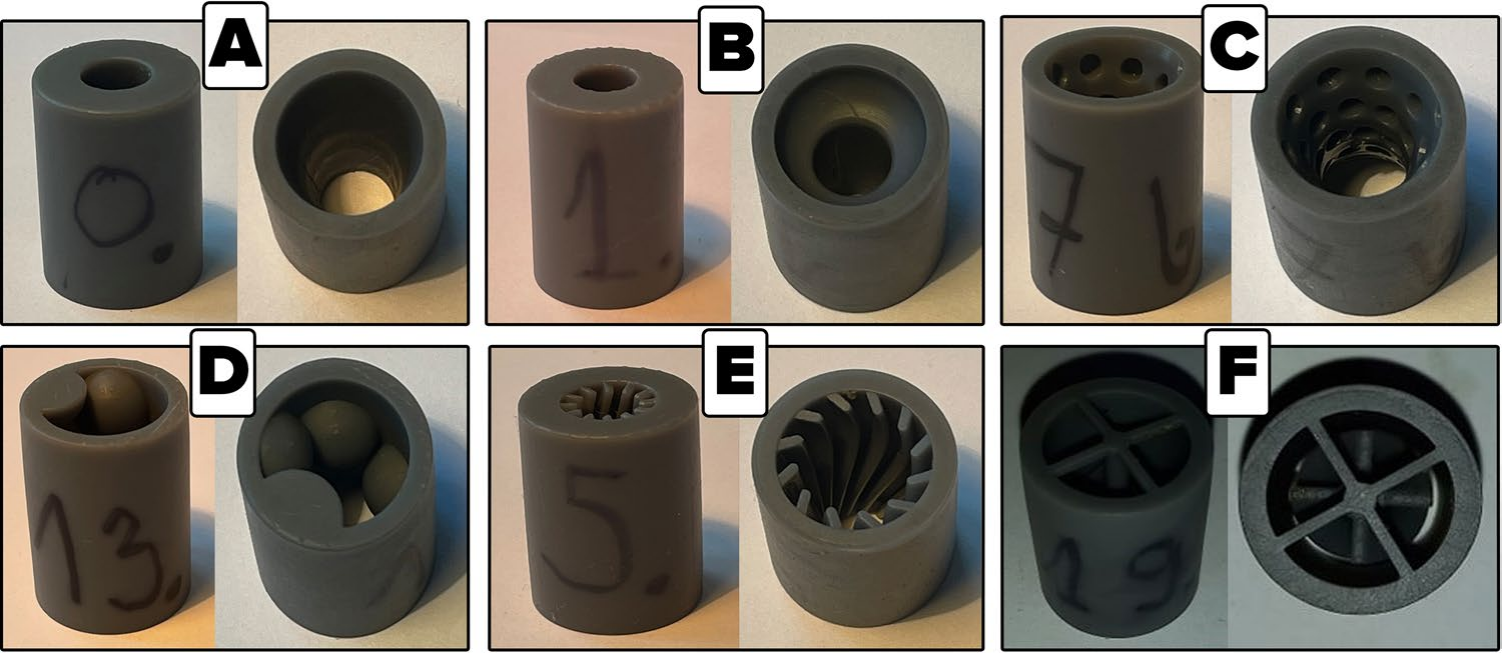
3D printed nozzles for reciprocating compressor manifold. (**A**) hyperboloidal shape, (**B**) brass instrument mouthpiece shape, (**C**) double hyperboloidal shape with cavities, (**D**) circumferential hemispheres shape, (**E**) ribbed shape, (**F**) turbine-like shape.

Nozzles printed for this test stand, from standard resin are shown in the Fig. 3.

#### Calculation of the specific compression power for reciprocating compressor

**Figure 4:**
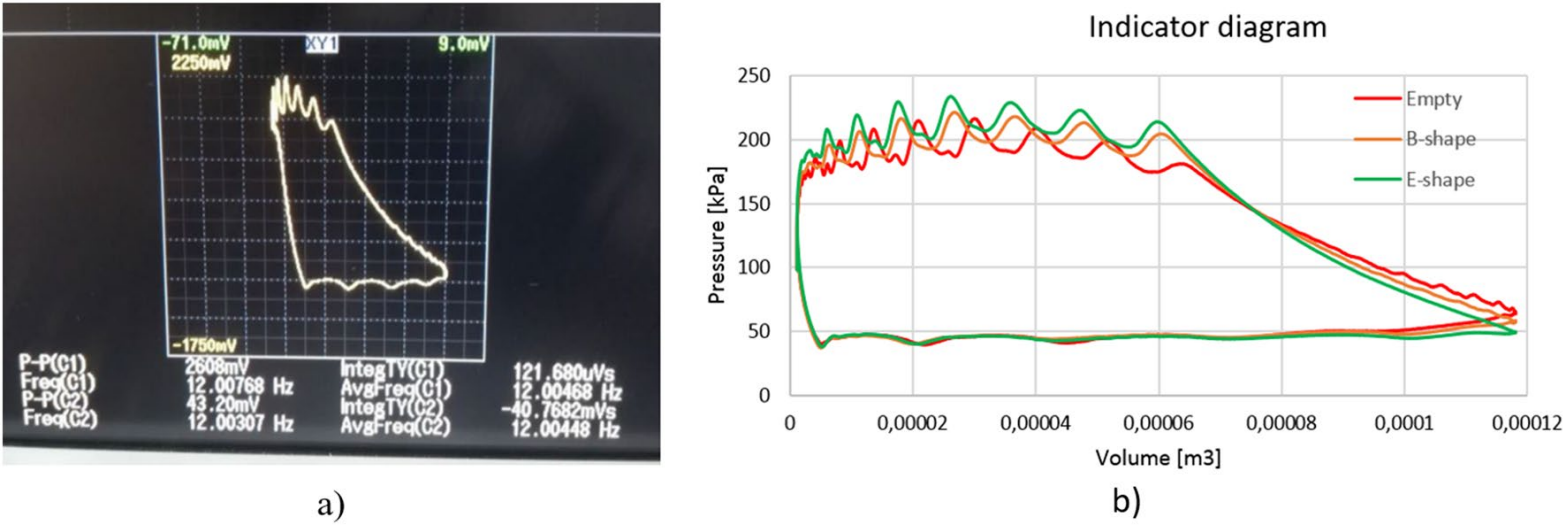
Indicator diagram for: (**a**) the reciprocating compressor on the oscilloscope (**b**) for the calculation of the compression power from oscilloscope data for three cases.

In order to perform accurate calculations of the specific compression power, the compressor indicator diagram method was used. This is a very popular method often used in scientific work^20^. The power was obtained from the indicator diagram recorded by the electronic YOKOGAWA DLM202 oscilloscope, where the measured values are averaged values from the last 128 cycles. The current pulsation frequency was read from the diagram in Fig. 4a. The sensor for measuring fast-changing pressures was placed in the compressor cylinder which allowed for this type of measurement. The current position of the drive shaft was measured with an encoder.

**Figure 5:**
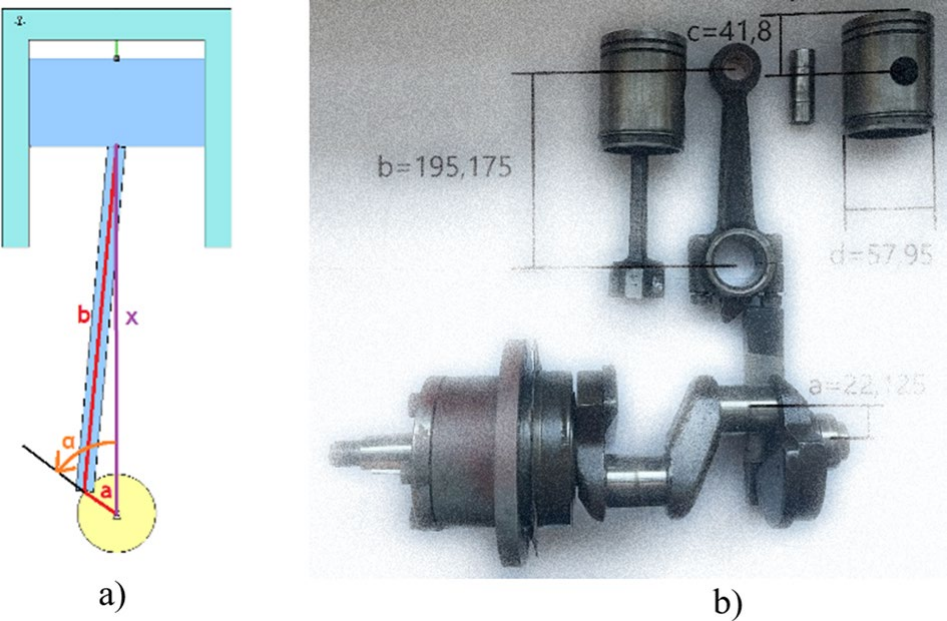
(**a**) A scheme of geometry of compressor components, where a – an eccentric on the crankshaft, b—connecting rod length, α – angle of rotation of the crankshaft (**b**) real compressor elements dimensions.

The compression power was calculated as the area between the curves from the indicator diagram. The Fig. 4b shows an indicator diagram for an installation with an empty nozzle and with two exemplary nozzles attached. Based on the geometry of the reciprocating compressor elements (Fig. 5), appropriate equations were derived to calculate the accurate volume above the piston.

For the assumed components geometry, a formula was developed to calculate the volume above the piston in relation to its exact position in the chamber. Formula (1) takes into account the clearance volume and the shaft rotation angle:

1 $$V\left(\alpha \right)=\pi \cdot {r}^{2}\left(a+b-\sqrt{{a}^{2}+{b}^{2}-2ab\cdot \mathrm{cos}(2\pi -\alpha -arcsin\frac{a\cdot \mathrm{sin}\alpha }{b}}\right)+{V}_{0}.$$

where:

${V}_{0}$—clearance volume, *V*—volume above the piston, *r*—piston radius, *a*—an eccentric on the crankshaft, *b*—connecting rod length, $\alpha$—angle of rotation of the crankshaft.

Determination of the volume for a given position of the drive shaft was the main obstacle in calculation of the indicated power. Compressor work value ${(W}_{i}$) for one degree revolution of the drive shaft was calculated from the formula (2):

2 $${W}_{i}=\frac{1}{2}*\left({V}_{i}-{V}_{i-1}\right)*\left({P}_{i}-{P}_{i-1}\right).$$

where *P* is the absolute pressure value for a given volume under the piston. Work for one cycle was defined as the sum of individual works measured every 1 degree (3):

3 $$\mathrm{W}=\sum_{i=0}^{i=360}{W}_{i}.$$

The exact value of the work is therefore the sum of the partial works counted every 1 degree of rotation. The indicated power *N* is then calculated from Eq. (4) correspondingly depending on the rotational speed of the drive shaft.

4 $$N=\mathrm{W}\cdot \frac{RPM{\prime}s}{60}$$

The value of the volume flow was determined on the basis of the parameters read on the thermoanemometer. The ratio of the volume flow to the compressor power, calculated with the above method, is the unit compression power for tests performed on the reciprocating compressor stand. After recalculating the volumetric flow rate to the standard conditions this quantity tells how much m^3^_nSI_ can be compressed with the usage of 1 kW of energy.

### Screw compressor–test stand

**Figure 6:**
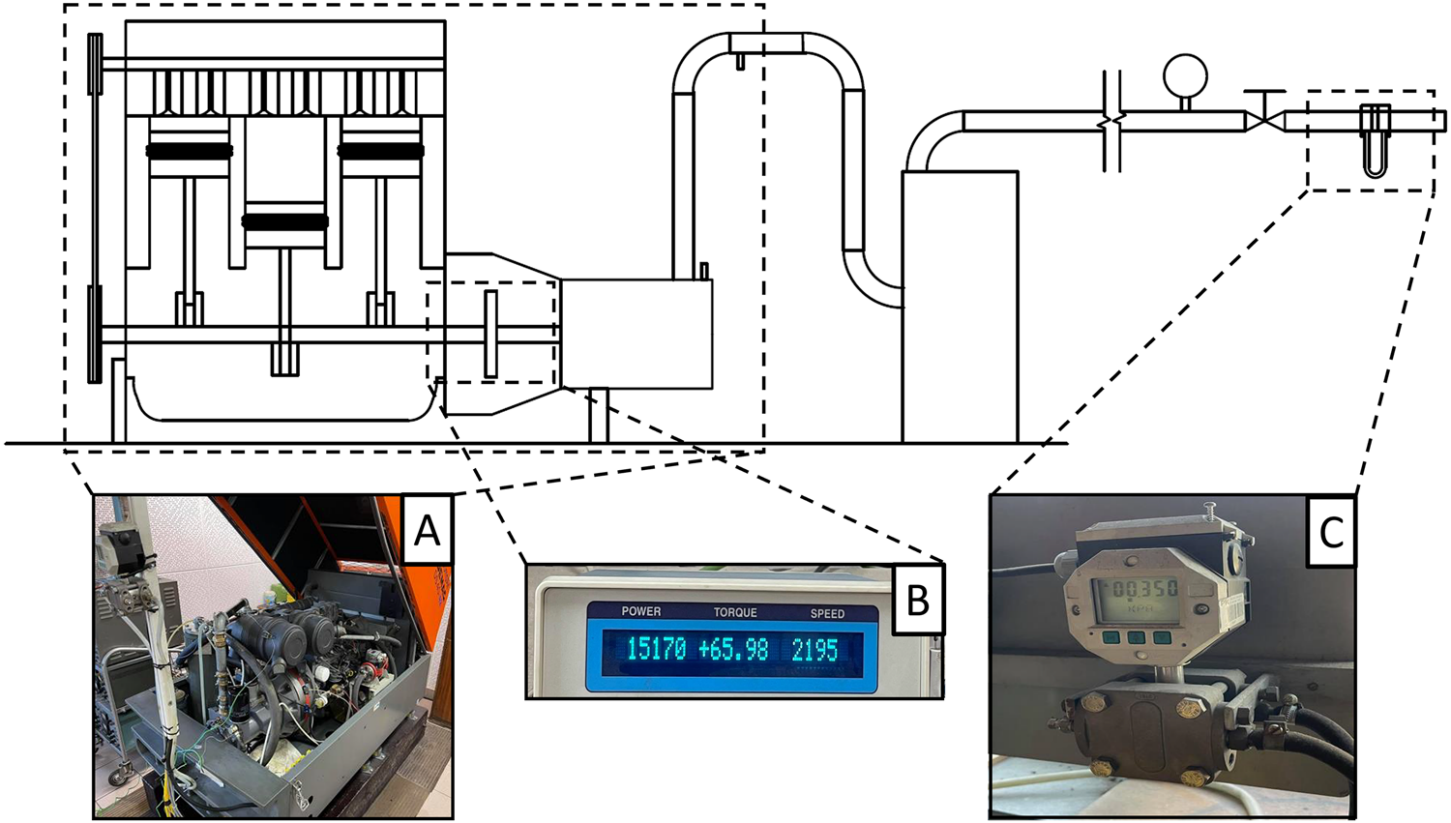
The scheme of the test stand with screw compressor. (**A**) Compressor unit: Compair DEMAG DS.40, (**B**) Magtrol 3410 Torque Display, (**C**) Differential pressure gauge GigibarII PE350.

The main element of the second test stand is the DEMAG.DS-40 CompAir compressor unit. A three-cylinder combustion engine with a power of 35.6 kW serves as the drive. This engine drives a screw compressor with a nominal capacity of 4.2 cubic meters per minute at an operating pressure of 700 kPa. Test stand scheme is shown in Fig. 6.

**Figure 7:**
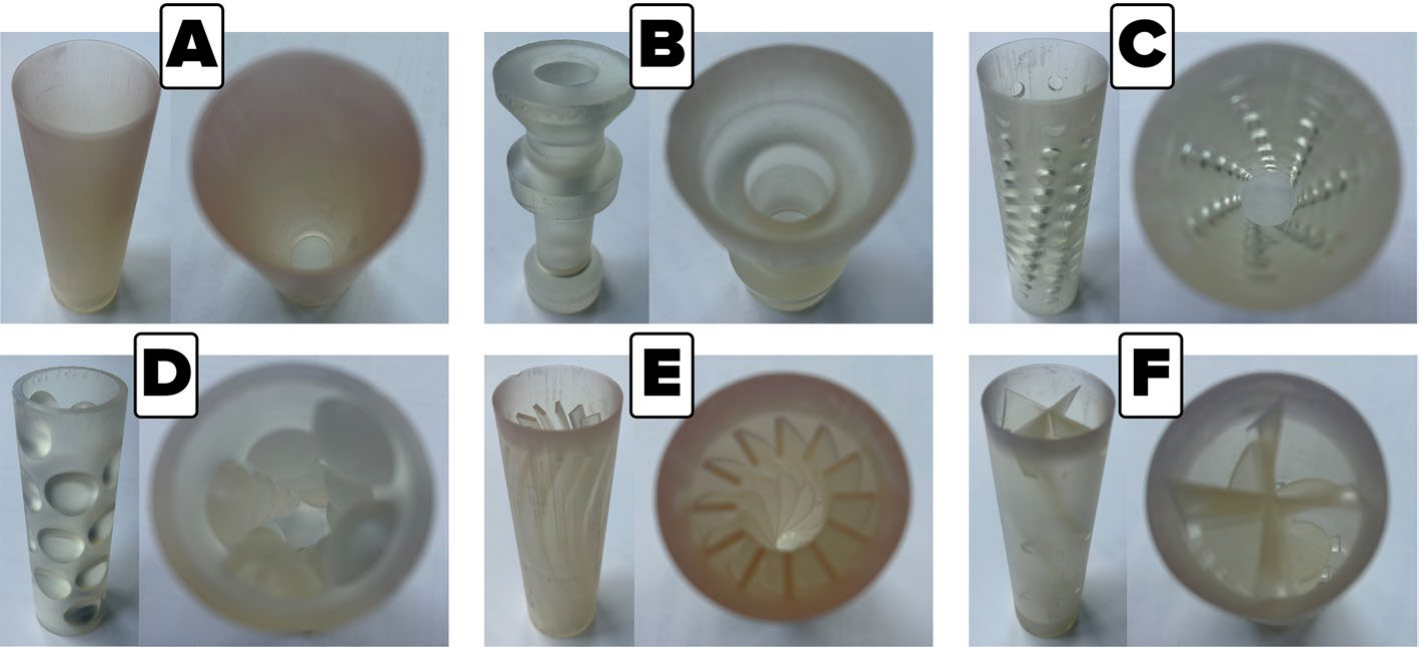
3D printed nozzles for screw compressor manifold. (**A**) hyperboloidal shape, (**B**) brass instrument mouthpiece shape, (**C**) double hyperboloidal shape with cavities, (**D**) circumferential hemispheres shape, E – ribbed shape, (**F**) turbine-like shape.

The discharge pressure was kept constant at 310 kPa during the research. For each case, the mass flow rate was calculated with the use of a metering orifice. The compression power was read from a Magtrol torque gauge with an accuracy of 0.01% for revolution speed readings and 0.01% for torque value. Compressor outlet pipe, installation pipe and nozzle mounting pipe have an inner diameter of 36 mm. The total length of the installation from the compressor to the oil separator tank is about 1 m long. Nozzles printed for this test stand, are shown in the Fig. 7. The B-shape nozzle presented in Fig. 7 was manufactured with the assumption of reducing the printing time and the amount of used material, with no effect on the assembly and gas flow through the nozzle. This nozzles are printed from high-temperature resin.

#### Calculation of the specific compression power for screw compressor

In the screw compressor installation, an accurate metering orifice (standardized orifice plate with parathyroid pressure) was used to determine the mass flow rate. Measurements of this type are specified by the PN-EN ISO 5167 standard^21^, which was used in the calculations presented in this part of the article. The power was read directly from the torque gauge. Specific compression power for the screw compressor test stand is defined as a the number of grams of gas that are compressed by 1 kW of energy.

## Results

The results of the influence of different nozzle shapes installed in the discharge manifolds of compressors were determined on the basis of the measurement data and calculation methods described in the previous chapter. Experiments were performed on both test stands in two independent series and the presented final results are the averaged values of two series. Accuracy of the measurements was calculated using the standard uncertainty indicator for complex measurand, as presented in the Eq. (5).

5 $$u\left(x\right)=\sqrt{{\left[\frac{\partial x}{\partial {x}_{1}}u({x}_{1})\right]}^{2}+{\left[\frac{\partial x}{\partial {x}_{2}}u({x}_{2})\right]}^{2}+\cdots +{\left[\frac{\partial x}{\partial {x}_{n}}u({x}_{n})\right]}^{2}}$$

For the discharge manifold in the reciprocating compressor installation, the power was calculated on the basis of the indicator diagrams. To determine the mass flow rate, a thermoanemometer was installed at the outlet of the tank in a pipe with flow area equal to 0.001225 m^2^. The indicator graph was generated as an averaged value from 128 cycles so it was assumed that values obtained from this measurement are accurate. Therefore, the uncertainty for a specific compression power, when only volumetric flow rate uncertainty was taken into account, was calculated from Eq. (6).

6 $$u\left(\frac{\dot{V}}{N}\right)=\frac{\partial \frac{\dot{V}}{N}}{\partial \dot{V}}u(\dot{V})=\frac{1}{N}u(\dot{V})$$

Uncertainty calculated this way obtains maximum value of 12% for the B shape for 720 rpm’s.

The results obtained for the analyzed nozzles in the reciprocating compressor installation are presented in Table 1. The results obtained for the measurements in the discharge manifold of the screw compressor are presented in Table 2.

**Table 1:** Results for a specific compression power loss in a reciprocating compressor test stand for different compressor shaft revolution speeds.

	RPM	Nozzle no						
		Empty	A	B	C	D	E	F
N [W]	720	301	322	311	324	323	323	320
	1080	482	526	511	514	519	510	500
$\dot{V}$[dm^3^_nsi_/s]	720	2.03 ± 0.21	1.91 ± 0.23	1.91 ± 0.23	1.92 ± 0.23	2.04 ± 0.24	2.04 ± 0.24	2 ± 0.24
	1080	2.71 ± 0.28	2.54 ± 0.27	2.2 ± 0.25	2.82 ± 0.30	2.78 ± 0.28	2.84 ± 0.28	3.07 ± 0.30
$\dot{V}$/N [dm^3^_nsi_/s/kW]	720	6.76 ± 0.71	5.93 ± 0.71	6.14 ± 0.74	5.94 ± 0.72	6.31 ± 0.73	6.3 ± 0.73	6.25 ± 0.74
	1080	5.62 ± 0.57	4.83 ± 0.51	4.30 ± 0.48	5.48 ± 0.58	5.37 ± 0.54	5.57 ± 0.55	6.14 ± 0.60
$\dot{V}$/N loss [%]	720	0	12.3	9.2	12.2	6.7	6.8	7.5
	1080	0	14.1	23.5	2.3	4.4	0.8	− 9.3
	avg	0	13.2	16.3	7.2	5.6	3.8	− 0.9

**Table 2:** Results for a specific compression power loss in screw compressor test stand test stand for different compressor shaft revolution speeds.

	RPM	Nozzle no						
		Empty	A	B	C	D	E	F
N [kW]	2200	14.07	14.59	14.96	14.75	14.51	14.9	14.29
	2000	12.55	12.91	13.49	13.39	12.97	13.21	12.89
	1800	11.5	11.65	11.88	11.78	11.55	11.77	11.39
	1600	9.7	10.35	10.25	10.3	10.18	10.17	10.21
$\dot{m}$[g/s]	2200	55.3	55.19	55.53	56.52	55.47	55.66	55.19
	2000	50.85	50.86	50.99	51.76	50.88	51.05	50.65
	1800	46.27	46.38	46.38	47.08	46.38	46.42	45.54
	1600	41.83	42.05	41.83	42.28	41.9	41.9	47.01
$\dot{m}$/N [g/s/kW]	2200	0.393 ± 0.0197	0.378 ± 0.0189	0.371 ± 0.0186	0.383 ± 0.0192	0.382 ± 0.0191	0.374 ± 0.0187	0.386 ± 0.0193
	2000	0.405 ± 0.0203	0.394 ± 0.0197	0.378 ± 0.0189	0.387 ± 0.0193	0.392 ± 0.0196	0.386 ± 0.0193	0.393 ± 0.0197
	1800	0.402 ± 0.0201	0.398 ± 0.0199	0.390 ± 0.0195	0.400 ± 0.0200	0.402 ± 0.0201	0.394 ± 0.0197	0.400 ± 0.0200
	1600	0.431 ± 0.0222	0.406 ± 0.0202	0.408 ± 0.0205	0.410 ± 0.0203	0.412 ± 0.0208	0.412 ± 0.0206	0.461 ± 0.0230
$\dot{m}$/N loss [%]	2200	0	3.8	5.5	2.5	2.7	5	1.7
	2000	0	2.7	6.7	4.6	3.1	4.6	3
	1800	0	1	3	0.7	0.2	2	0.6
	1600	0	5.8	5.4	4.8	4.6	4.5	− 6.8
	avg	0	3.3	5.2	3.1	2.7	4	− 0.4

Although the uncertainty of the measurements performed on the reciprocating compressor manifold may appear high, the averaged values calculated from two independent experiments allow us to read the general trends. The gain in SCP for the F-shape can be explained by the phenomenon of dynamic charging, which is widely reported in the literature. Due to the accuracy range, this value can be positive, but only slightly, which is also important information. Because of the relatively high inaccuracies obtained on the reciprocating compressor bench, tests were also performed on the screw compressor bench. This approach allowed for verifying the results from the piston compressor test stand and assessing the phenomenon of scalability of the solution.

In the case of a screw compressor installation, the power was measured by a torque gauge mounted on the compressor drive shaft. Power is calculated by the torque gauge based on the measurements of torque (T) and shaft revolution speed (ω). When revolution speed is measured in rpm then formula (7) is used:

7 $$N=T\cdot \frac{\omega }{9.5488}$$

and uncertainty is calculated from Eq. (8):

8 $$u\left(N\right)=\sqrt{{\left[\frac{\partial N}{\partial T}u(T)\right]}^{2}+{\left[\frac{\partial N}{\partial \omega }u(\omega )\right]}^{2}}=\sqrt{{\left[\frac{\omega }{9.5488}\cdot u(T)\right]}^{2}+{\left[\frac{T}{9.5488}\cdot u(\omega )\right]}^{2}}$$

Uncertainties obtained this way for all measurements are less than 0.002 kW which is less than 0.01% for all measurements. Thus they will not be included in the table as negligible.

Measurement of the mass flow of the compressed gas was calculated based on the readings from the metering orifice, in accordance with PN-EN ISO 5167 standard. The standardized orifice plate with parathyroid pressure reception was used as the metering orifice. Based on the research done by^22^, the measurement uncertainty of the mass flux for this type of nozzle with constriction β = 0.4 is maximum 0.5% of the measured value. Therefore the maximum (for the worst case scenario) uncertainty for the complex expression $\dot{m}$/N was calculated, from Eq. 9., and shown in the table as important.

9 $$u\left(\frac{\dot{m}}{N}\right)=\sqrt{{\left[\frac{\partial \frac{\dot{m}}{N}}{\partial N}u(N)\right]}^{2}+{\left[\frac{\partial \frac{\dot{m}}{N}}{\partial \dot{m}}u(\dot{m})\right]}^{2}}=\sqrt{{\left[-\dot{\frac{m}{{N}^{2}}}\cdot u(N)\right]}^{2}+{\left[\frac{1}{N}\cdot u(\dot{m})\right]}^{2}}$$

The resultant uncertainty for the SCP is for all cases around or less than 0.02 [g/s/kW].

The SCP for tested shapes ranges from values close to zero to about 5% for the nozzle mounted in the discharge manifold of a screw compressor. Taking into account the uncertainties it can be assumed that introducing the nozzles into the screw compressor manifold the influence on the SCP may be acceptable and have no significant impact on the efficiency of operation of such an installation. For tests performed on the reciprocating compressor test stand, the differences are much higher, and the specific compression power, in extreme cases, increases by 16% in relation to the empty manifold.

**Figure 8:**
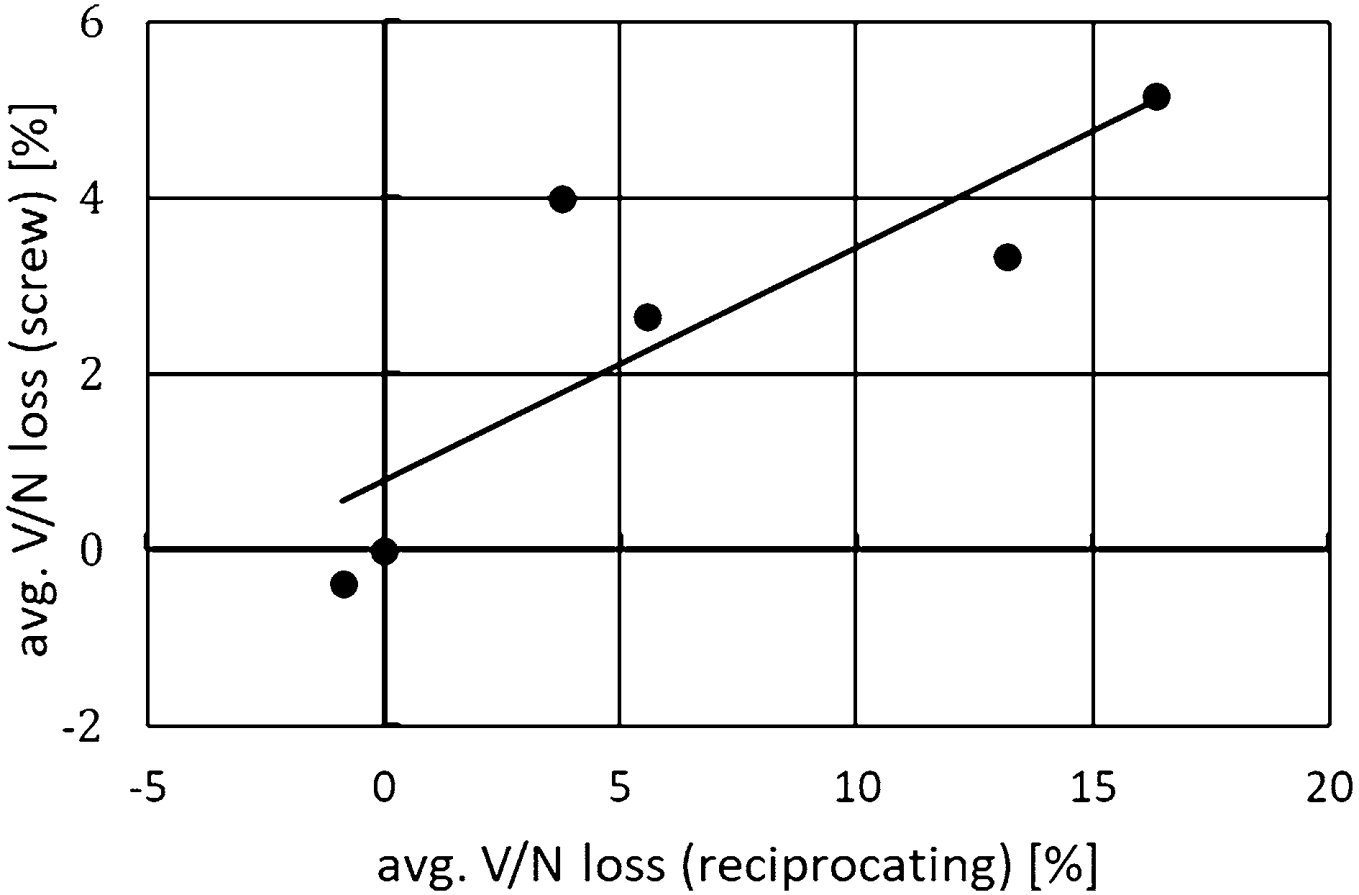
Averaged V/N loss values comparison obtained on reciprocating and screw compressor test stands.

The comparison of the averaged results obtained from both test stands is shown in Fig. 8. The calculated correlation R^2^ is close to 0.7, which may indicate a certain repeatability of the influence of shapes on the compression power in different environments.

### Power demand predictive model

The main reason for the increase in the demand for compression power with the use of flow constriction nozzles is the local pressure losses generated on the nozzle. In the case of pressure pulsations, there are also acoustic phenomena which have a different influence on the compression power. Due to the complex shapes of the designed nozzles determination of the pressure loss generated by them is complicated. One possible solution is CFD calculation for a given nozzle shape. However, it is a procedure that requires specialized skills, software and time. Therefore, in the next part of this article, a simple method of determining the influence of nozzles on the demand for compression power is presented.

**Figure 9:**
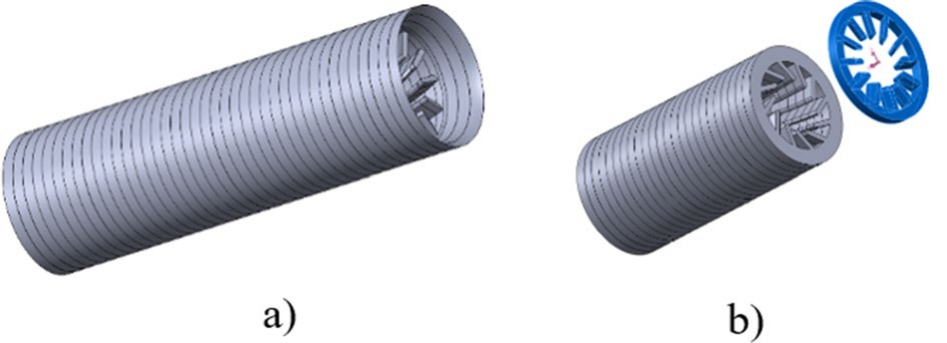
Nozzle CAD model for calculations. (**a**) Nozzle divided into slices (**b**) a single slice against the rest of the nozzle.

In CAD software, the nozzle was cut into slices (Fig. 9). Thereafter, the volume of each new formed slice was read, which was then divided by the height of the slice. This is how an average cross-sectional area for a given new-formed slice was obtained. The comparison of the average cross-sectional area of the slice to the cross-sectional area of the pipeline made it possible to calculate the percentage of flow constriction. For some geometries (e.g. axially symmetrical), a simpler method can be used, but the described calculation method can be applied to all, even the most complicated, nozzles geometries. The accuracy of the calculated cross-sectional area depends on the number of slices cut off from the nozzle. In this calculation the nozzle, for the screw compressor manifold, was cut into 40 slices (Fig. 9a). Each slice was 3 mm high (Fig. 9b).

For each extracted nozzle slice, the pressure drop coefficient (${\Pi }_{\Delta p,i}$) was calculated from the simplified form of the formula (10) used to determine the pressure drop in gas pipelines with a circular cross-section:

10 $$\Delta p=\lambda \cdot \frac{L}{D}\cdot \frac{\rho }{2}\cdot {w}^{2}.$$

For the comparative analysis, it was assumed that the coefficient *λ*, density *ρ* and flow velocity *w* are the same for all nozzles. Taking into account the same constriction value for all analyzed nozzles, this simplification was accepted due to the generation of small differences in the average flow velocity. The coefficient *Π*_*Δp,i*_ obtained in this way determines the pressure drop on individual elements and is determined by the Eq. (11):

11 $${\Pi }_{\Delta p,i}=\frac{{L}_{i}}{{D}_{eqv,i}}.$$

where L_i_ is the length with the area value with consecutive constrictions and *D*_*eqv*_ is an equivalent diameter for consecutive flow area constrictions: 80%, 60%, 40% and 30%, calculated from Eq. (12) :

12 $${D}_{eqv,i}=\sqrt{\frac{{4\cdot A}_{i}}{\pi }}.$$

Where *A*_*i*_ is consecutive flow area constriction: *i* = 1 for 80%, *i* = 2 for 60%, *i* = 3 for 40% and *i* = 4 for 30%.

As a result of the calculations, 4 pressure drop coefficients were obtained for each nozzle. The sum of these coefficients is the total pressure drop coefficient ($\sum_{i}{\Pi }_{\Delta p,i}$) for each shape. The values obtained for the tested nozzles at both test stands are presented in Table 3.

**Table 3:** The value of the ΠΔp parameter for the nozzles relative to the value of the Pi parameter for an empty pipe.

Nozzle no	Reciprocating compressor manifold									
	L_1_	L_2_	L_3_	L_4_	${\Pi }_{\Delta p,1}$	${\Pi }_{\Delta p,2}$	${\Pi }_{\Delta p,3}$	${\Pi }_{\Delta p,4}$	$\sum_{i}{\Pi }_{\Delta p,i}$	$\sum_{i}{\Pi }_{\Delta p,i}$ gain [%]
Empty	0.03	0	0	0	1.696	0	0	0	1.458	0
A	0.005	0.007	0.011	0.008	0.297	0.441	0.84	0.692	1.951	33.8
B	0.005	0.001	0.001	0.023	0.297	0.049	0.06	2.147	2.194	50.5
C	0.008	0.011	0.011	0	0.424	0.734	0.9	0	1.769	21.3
D	0.017	0.014	0	0	0.933	0.881	0	0	1.56	7
E	0.012	0.014	0.005	0	0.678	0.881	0.36	0	1.65	13.2
F	0.029	0.001	0	0	1.654	0.049	0	0	1.464	0.4

Nozzle no	Screw compressor manifold									
	L_1_	L_2_	L_3_	L_4_	${\Pi }_{\Delta p,1}$	${\Pi }_{\Delta p,2}$	${\Pi }_{\Delta p,3}$	${\Pi }_{\Delta p,4}$	$\sum_{i}{\Pi }_{\Delta p,i}$	$\sum_{i}{\Pi }_{\Delta p,i}$ gain [%]
Empty	0.12	0	0	0	3.833	0	0	0	3.833	0
A	0.039	0.018	0.036	0.027	1.246	0.664	1.626	1.408	4.944	29
B	0.018	0.003	0	0.099	0.575	0.111	0	5.164	5.85	52.6
C	0.048	0.036	0.036	0	1.533	1.328	1.626	0	4.487	17.1
D	0.030	0.09	0	0	0.958	3.32	0	0	4.278	11.6
E	0.012	0.042	0.051	0.015	0.383	1.549	2.304	0.782	5.019	30.9
F	0.105	0.015	0	0	3.354	0.553	0	0	3.907	1.9

**Figure 10:**
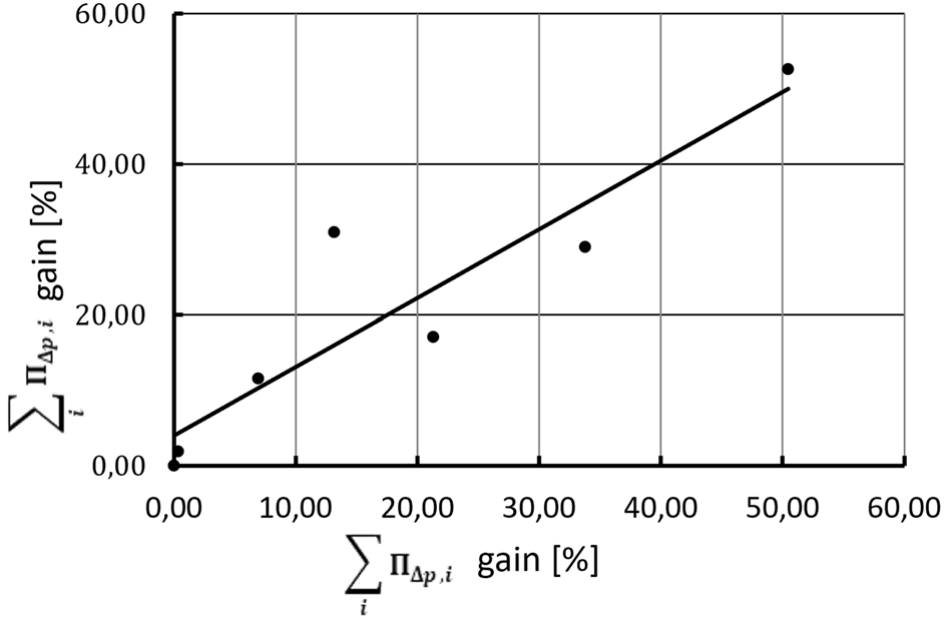
Cumulative values of the sum coefficients comparison obtained for both test stands.

In Fig. 10 it can be seen that the total values of the coefficients for the same nozzles in different installations are more strongly correlated than the increase in the specific compression power shown in Fig. 8, as the correlation coefficient is *R*^*2*^ = 0.8416. This is not surprising as the model operates only on one variable (the nozzle slices flow area), and many other factors appear in the experimental investigations.

**Figure 11:**
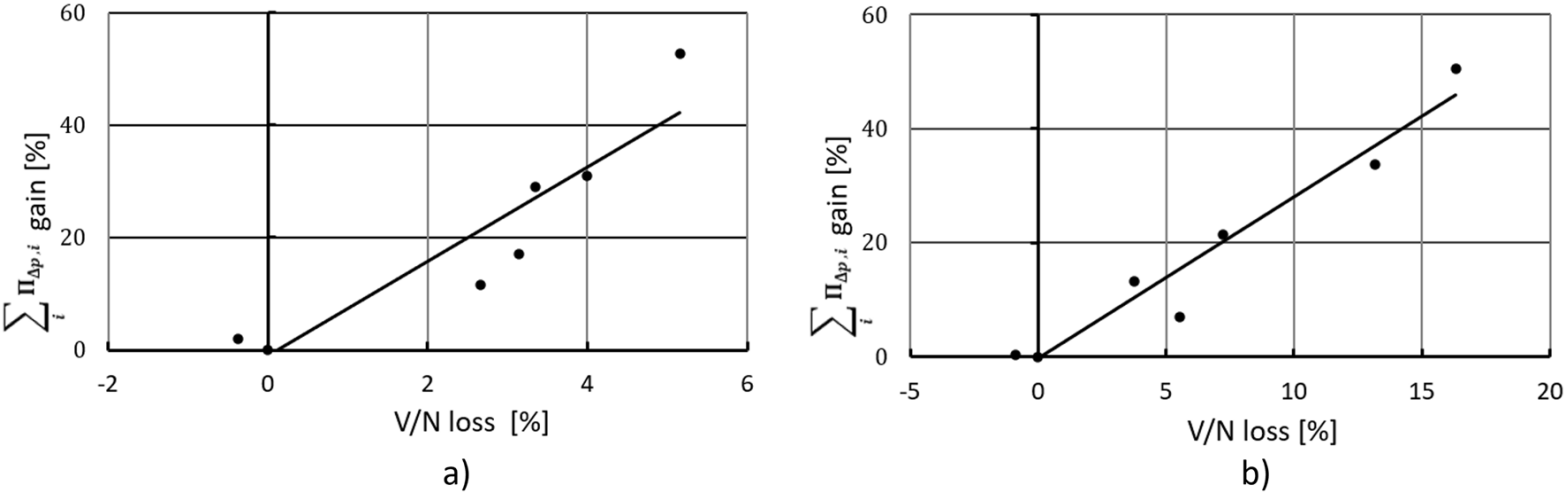
Modeled vs obtained influence of a nozzle on the compression power in: (**a**) screw compressor manifold and (**b**) reciprocating compressor manifold.

The most important purpose of the presented simplified model for assessing the impact of a nozzle on the specific compression power is the ability to determine the influence of a given nozzle shape on the actual compression efficiency characteristics, which is shown in Fig. 11.

From results shown in Fig. 11 it can be seen that the proposed simple model for determining the influence of a different shape nozzles, on the increase of the specific compression power, works well for the tested elements. The Pearson coefficient *R*^*2*^ is equal to *R*^*2*=^0.8478 for screw compressor test stand and *R*^*2*=^0.9419 for reciprocating compressor manifold.

## Conclusions

The article presents an assessment of the impact of SNT on the specific compression power, which defines the energy efficiency of the compression process. The tested nozzles were characterised by a high constriction of the cross-section of the pipeline (70%), which allow to highlight the differences generated by different shapes. The obtained results are promising because it is shown that even for large values of the constriction, the averaged decrease in the efficiency of the compression process, in relation to the empty pipe in the discharge manifold of the screw compressor, ranges from about 0 to about 5%. The differences are much more significant and range from approx. 0 -16% in the case of lower frequencies in the manifold of a reciprocating compressor. It is important to note that for almost every nozzle, these values dropped below 2% for the rotational speed (*RPM* = *1800*) of the screw compressor drive shaft. It means that it is possible to design an element, for a particular pulsation frequency, with an acceptable effect on the compression power in relation to the benefits obtained from the damping of pulsations.

The research showed that the influence of the tested elements on the compression power was much lower in the case of the screw compressor installation. Perhaps this is the place to look for applications for the presented SNT technology.

The measurement uncertainties obtained in the reciprocating compressor test stand for individual experiments may seem large. However, in the case of comparative analysis this is not crucial and the averaged values seem to correspond with those obtained on the screw compressor bench, where very high test accuracy was obtained.

The article also presents a simple method that allows to estimate the influence of nozzles with complicated shapes on the demand for compression power. From the obtained results, it can be concluded that the method effectively predicts this influence. The accuracy of the method depends on the number of slices into which the nozzle CAD model is divided. However, it should be remembered that the model is based only on the calculation of the quantity related to flow resistance, and in the real installation there may also be acoustic phenomena and flow character-related phenomena.

In summary, the work presented in the article shows that the installation of the shaped nozzles in a pipeline could not significantly, or at all, affect the consumption of energy necessary to compress the gas. Further research should be carried out to prove that SNT is safe in operation of gas pipelines, and sufficiently effective in damping pressure pulsations and pipeline vibrations.

## Data Availability

The datasets used and/or analysed during the current study available from the corresponding author on reasonable request.

## List of symbols

$\alpha$: Angle of rotation of the crankshaft [º]
${\Pi }_{\Delta p,i}$: Pressure drop coefficient [-]
*a*: An eccentric on the crankshaft [m]
*b*: Connecting rod length [m]
D: Pipeline diameter [m2]
$${D}_{eqv,i}$$: Equivalent diameter [m]
L_i_: Slice length [m]
$\dot{m}$: Mass flow rate [g/s]
N: Power [W] and [kW]
*r*: Piston radius [m]
u(x): Uncertainty of x-measurement value [proper for value]
W: Work [J]
$${V}_{0}$$: Clearance volume [m3]
*V*: Volume above the piston [m3]
$\dot{V}$: Volumetric flow rate in standard conditions [m3nsi/s]
